# Prefrontal Neural Activity When Feedback Is Not Relevant to Adjust Performance

**DOI:** 10.1371/journal.pone.0036509

**Published:** 2012-05-16

**Authors:** Jale Özyurt, Mareike Rietze, Christiane M. Thiel

**Affiliations:** 1 Biological Psychology Lab, Institute of Psychology, Carl von Ossietzky Universität, Oldenburg, Germany; 2 Research Center Neurosensory Science, Carl von Ossietzky Universität, Oldenburg, Germany; University College London, United Kingdom

## Abstract

It has been shown that the rostral cingulate zone (RCZ) in humans uses both positive and negative feedback to evaluate performance and to flexibly adjust behaviour. Less is known on how the feedback types are processed by the RCZ and other prefrontal brain areas, when feedback can only be used to evaluate performance, but cannot be used to adjust behaviour. The present fMRI study aimed at investigating feedback that can only be used to evaluate performance in a word-learning paradigm. One group of volunteers (N = 17) received informative, performance-dependent positive or negative feedback after each trial. Since new words had to be learnt in each trial, the feedback could not be used for task-specific adaptations. The other group (N = 17) always received non-informative feedback, providing neither information about performance nor about possible task-specific adaptations. Effects of the informational value of feedback were assessed between-subjects, comparing trials with positive and negative informative feedback to non-informative feedback. Effects of feedback valence were assessed by comparing neural activity to positive and negative feedback within the informative-feedback group. Our results show that several prefrontal regions, including the pre-SMA, the inferior frontal cortex and the insula were sensitive to both, the informational value and the valence aspect of the feedback with stronger activations to informative as compared to non-informative feedback and to informative negative compared to informative positive feedback. The only exception was RCZ which was sensitive to the informational value of the feedback, but not to feedback valence. The findings indicate that outcome information per se is sufficient to activate prefrontal brain regions, with the RCZ being the only prefrontal brain region which is equally sensitive to positive and negative feedback.

## Introduction

Whenever information is ambiguous referring to action outcomes, external feedback provides essential information needed for performance evaluation and subsequent adaptation. Brain mechanisms of external positive- and negative-feedback processing have been examined in numerous studies using functional magnetic resonance imaging (fMRI), electroencephalography (EEG) and monkey electrophysiology, but results have partly remained inconsistent. fMRI studies have shown that anticipation and receipt of positive feedback mainly and quite consistently activates the striatum, even when non-monetary abstract performance feedback is used [1]–[4]. The medial and lateral parts of the orbitofrontal cortex, in line with their role in value calculation and value-based decision-making, have often been shown to be involved with positive and negative utilitarian feedback information, i.e. monetary gains and losses [1], [5]–[10]. Negative feedback has further been associated with heightened neural activity in several other medial and lateral prefrontal regions, but the presence of those activation foci considerably differed across studies. Activated clusters mainly included the rostral cingulate zone (RCZ) in the more posterior part of the anterior cingulate cortex (ACC), the pre-supplementary motor area (pre-SMA) and the lateral prefrontal cortex (inferior frontal cortex and insulae) [3], [5]–[6], [10]. Noteworthy, some studies reported the opposite pattern, i.e. stronger neural activity for positive compared to negative feedback in and mostly anterior to the RCZ [1]–[2], [4], [11].

Recent research has begun to shed some light on these inconsistent findings. For instance, it was demonstrated that partly similar fronto-subcortical networks are involved in the processing of both positive and negative feedback [12]–[16]. Further, similar neural activation to positive and negative feedback was observed in dorsal ACC (dACC)/RCZ in conditions, where both were of equal relevance for subsequent behavior [13], [17]–[18]. Finally, a recent time estimation study by Mies et al. [18] suggests that the RCZ is specifically driven by the validity of the feedback, irrespective of its valence, provided that positive and negative feedback are of equal importance for subsequent behaviour.

Overall, there is now accumulated evidence for a role of the RCZ to evaluate the relevance of positive and negative feedback in view of upcoming task performance and to accordingly fine tune the behaviour for the next trial [11], [18]. Moreover, positive feedback-related activation in the dACC/RCZ has been proposed to be generally dependent on its relevance i.e., whether it bears information for a correct response in the following trial [19]. In line with this, valence insensitivity of the RCZ, due to a similar activation to positive and negative feedback, has only been shown in studies where both feedback types were of equal relevance and additionally allowed for performance adjustments in the next trial [17]–[18], [20]. Less is known, however, about how the brain and the RCZ in particular, processes positive and negative feedback when these are not relevant for such adjustments. It is well conceivable that valence insensitivity may only occur in situations where both feedback types can be used to fine tune task-specific behavior in the next trial.

Accordingly, the present study aimed to investigate the sensitivity of the RCZ and other prefrontal areas to informative feedback and feedback valence when feedback cannot be used to adjust performance in the following trial. We used a task resembling language learning, where subjects had to learn new words in each trial and received feedback to evaluate current performance. Feedback that follows a single learning trial and just gives information about success or failure is present in many real-life interactions. To assess effects of the informative value of feedback, we employed a between-subject design, where one group of volunteers received positive and negative feedback depending on performance and the other group always received non-informative feedback, independent of performance. To assess effects of feedback valence, a within-subject comparison between positive and negative feedback was performed in the group receiving informative feedback. If valence insensitivity only occurs in situations where both, positive and negative feedback can be used for adjustments in upcoming performance this analysis should reveal differential activity in RCZ as in other prefrontal areas to both types of feedback.

## Methods

### Subjects

36 right-handed adult healthy volunteers took part in the experiment. All subjects had normal or corrected-to-normal vision and normal hearing abilities. The study was conducted in accordance with the Declaration of Helsinki and all procedures were carried out with the adequate understanding and written consent of the participants. Ethics approval was obtained from the ethics committee of the Carl von Ossietzky University. After the scanning session two participants were excluded from further analysis, one due to excessive head movements (displacement with regard to the reference scan exceeded 3 mm) and another due to a low error rate (<4 trials). Thus, data from 34 volunteers (informative/non-informative feedback group: each N = 17, mean age: 24/23 yrs., SD = 2.4/2.2, 7/9 females) were included in the further analysis.

### Design and Experimental Paradigm

**Figure 1:**
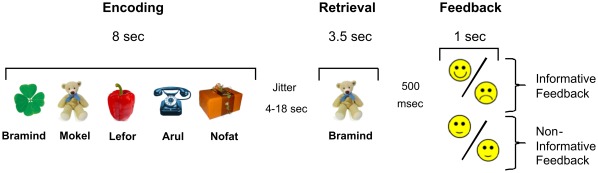
Time course of stimulus presentation in the word-learning task. Each trial started with an encoding phase consisting of 5 successively presented name-object pairs. Simultaneous with each object presentation a pseudoword, serving as object name, was presented via headphones. Subjects were instructed to memorize the name of each object. Recognition performance was tested after a jittered delay period and involved the presentation of one of the objects with one of the pseudowords. Participants had to indicate whether the pseudoword matched the object presented and responded on two buttons of a response pad (right index for correct and middle finger for incorrect). Feedback was provided immediately. In the informative group, feedback depended on performance, indicating either success or failure (positive or negative smiley). In the non-informative group, feedback was not dependent on performance. A neutral smiley appeared after each trial, giving no indication on success or failure.

We used a word learning paradigm which resembled learning vocabulary in a foreign language and involved learning arbitrary name-object associations (see Fig. 1). Task difficulty was piloted in an independent sample of subjects and adjusted to yield an average error rate of approximately 35%. In a between subjects design, volunteers were randomly assigned to two groups, both performing the same task. One group (informative feedback) consistently received performance-dependent positive and negative feedback after retrieval of a name-object association, the other group (non-informative feedback) received performance independent neutral feedback immediately after each trial. The two groups did not differ significantly with respect to age, sex and reasoning-based intelligence as tested with a German version of the Raven’s Standard Progressive Matrices [21]. A between subject design was chosen to compare a situation where subjects were either used to always receiving informative feedback or non-informative feedback, rather than intermixing those two types of feedback within subject.

Each trial involved an encoding phase lasting for 8 seconds and consisting of 5 successively presented name-object pairs. Each object was presented for 1.2 sec with an inter-stimulus interval of 400 msec. Simultaneous with each object presentation, a meaningless but phonologically regular disyllabic pseudoword, serving as object name, was presented via MR-compatible headphones (MR-Confon GmbH, Magdeburg, Germany). Subjects were instructed to memorize the name of each object. Recognition performance was tested immediately after a jittered delay period (4 to 18 sec, mean 8 sec) and involved the presentation of one of the objects with one of the pseudowords for 1.5sec. Participants had to indicate within 3.5 sec (from presentation on) whether the pseudoword was the correct or incorrect object name. They responded with the right index and middle finger on two buttons of a fiber-optical MRI-compatible response pad (LUMItouch system; Photon Control, Burnaby, British Columbia, Canada) placed on the right side of their body. A button press with the index finger of the right hand indicated a correct and a button press with the middle finger indicated an incorrect assignment, respectively. Feedback was provided after a short delay of 0.5 sec for duration of 1 sec. The informative feedback group always received a positive or negative smiley, indicating either success or failure in the present trial. The non-informative feedback group always received a neutral smiley, with no indication about success or failure. In case of a late or missed response an image of a clock was shown instead of a smiley in both groups. The response deadline used, together with a distinct feedback for response omissions or late responses, largely ruled out the possibility that subjects did not attend to the non-informative feedback. Additionally, we performed between-group comparisons of BOLD-activation in posterior brain regions known to be sensitive for modulations of visual attention (see section: fMRI data analyses). No significant between-group differences were obtained in occipital, temporal and parietal cortices, indicating a comparable attention deployment to informative and non-informative feedback stimuli.

The total time of each trial was varied between 17 and 31 sec. Further, a similar jitter as used for the delay period was applied for the inter-trial interval where subjects were instructed to maintain fixation on a central fixation cross. A total of 200 stimulus pairs were presented in each of the two experimental sessions but none of the pairs was presented twice. Thus, since new name-object associations were learnt in each trial, negative or positive feedback in our task merely provided outcome information that could be used to evaluate performance and increase the focus on task demands in case of errors. To keep total scanning time below 30 minutes for potential use of the task in children, the total number of trials was restricted to 40, presented in two consecutive sessions per 20 trials. Each name-object pair was only presented once. The order of the name object pairs presented in the encoding phase was pre-randomized and was the same for each of one subject out of the two feedback groups. Immediately after the scanning sessions, subjects filled in a self-made questionnaire, e.g. to estimate their own error rates (in percent). A training session with two runs in a mock scanner was performed one or two days prior to scanning to ensure proficiency in the task. For the training session we employed a similar task as used during scanning, differing solely in the stimulus categories presented (face-name associations). Due to the detailed practice session, subjects in the non-informative feedback group were well familiar with the condition lacking any informative feedback, thus ruling out expectancy effects that might have resulted in i.e. frustration. Both groups were also familiar with all other modalities of stimulus presentation, e.g. that each item was only presented once.

### MRI Procedure

A SONATA MRI system (Siemens, Erlangen, Germany) operating at 1.5 T was used with a standard whole-head coil to obtain T2*-weighted echoplanar (EPI) images with BOLD contrast (matrix size: 64×64, pixel size: 3×3 mm^2^, field of view: 192 mm). The experimental control software was programmed using Cogent 2000 (www.vislab.ucl.ac.uk/Cogent). Stimuli appeared on a back-projection screen mounted inside the scanner bore. Subjects could see the stimuli via a mirror attached to the head coil. 480 T_2_*-weighted gradient echo planar imaging (EPI) volumes with BOLD contrast were acquired (time to repeat (TR) = 2.5 sec; time to echo (TE) = 55 msec; flip angle α = 90°). These volumes consisted of 35 three mm-thick axial slices which were acquired sequentially with a 0.6 mm gap. Each volume covered the whole brain with the exception of the lower part of the cerebellum. A high resolution T1-weighted scan (176 contiguous slices, each slice 224×256 voxels, voxel size = 1×1×1 mm^3^) was conducted with a magnetisation prepared rapid acquisition gradient echo sequence (MPRAGE, TR = 1.97 s, TE = 3.93 ms and α = 15°) to collect a high-resolution structural volume of each participant.

### fMRI Data Analysis

Data were preprocessed and statistically analysed using the Statistical Parametric Mapping software SPM5 (Wellcome Department of Imaging Neuroscience, London). After spatial realignment and unwarping, the time series of each voxel was temporally realigned to the middle slice to correct for differences in slice acquisition time. Structural and functional volumes were coregistered and spatially normalised to a standard T1 template based on the MNI reference brain (resampled to 2×2×2 mm^3^ voxel). The data were then smoothed with a Gaussian kernel of 8 mm full-width-half-maximum to accommodate inter-subject anatomical variability.

At the single subject level, four regressors were entered into the design matrix. The first regressor modelled the encoding phase, regressors two and three coded for feedback presentation, depending on correct or incorrect performance. A fourth regressor of no interest coded for missed responses. Depending on the duration of the respective event, the regressors were convolutions of a box-car (encoding, duration 8 sec) or stick function (feedback) with a canonical synthetic haemodynamic response function time-locked to onsets of the respective events. The time series in each voxel was high-pass filtered to 1/128 Hz and modelled for temporal autocorrelation across scans with an AR(1) model.

At the group level, we used a mixed-effects model, focussing on neural activity during the feedback phase. For each subject of both groups, two weighted contrasts were entered into a two-way full-factorial ANOVA model. These contrasts coded for the fMRI signal increase with feedback presentation, i.e. performance dependent positive and negative feedback in the informative feedback group and performance independent neutral feedback (after correct responses and errors, respectively) in the non-informative feedback group.

**Figure 2:**
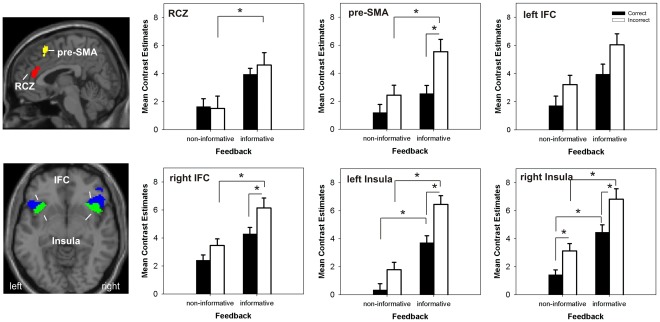
Activation results for the prefrontal regions of interest obtained from the factorial model. ROIs are shown overlaid on a single subject template included in SPM5 (left). The graphs on the right side show the mean activation profiles depicted for 6 ROIs in the rostral cingulate zone (RCZ, center of mass: x = 4 y = 37 z = 20), pre-supplementary motor area (pre-SMA, center of mass: x = 6 y = 20 z = 58), inferior frontal cortex (IFC, center of mass, left: x = −42 y = 21 z = −11; right x = 46 y = 23 z = 4) and anterior insulae (center of mass left: x = −35 y = 16 z = −10, right: x = 38 y = 18 z = −10). Bars represent mean activity averaged over the depicted respective ROI. Error bars depict the standard error of the mean. Black bars: correct performance, white bars: incorrect performance. *p<.008 (correction for 6 ROIs).

As we were mainly interested in feedback-related neural activity in prefrontal brain regions we chose a ROI approach and restricted the analyses to functional ROIs within anatomically predefined brain areas. For that purpose, the following prefrontal regions which were previously shown to be associated with negative and positive feedback processing, respectively, were selected from the Marsbar AAL ROI-Library [22], using the Marsbar toolbox [23]: cingulate cortex (anterior and middle, combined), supplementary motor area, insulae and inferior frontal cortices (IFC; pars orbitalis and triangularis, combined). These anatomical regions were combined (per intersection) with functional activation maps derived from the F-contrast of the effects of all conditions (i.e. regions whose activation significantly differed from baseline when summing across correct and error trials in both groups at p<.0001, FDR-corrected, extend threshold = 20 voxel). The sizes of the six ROIs generated with that procedure are depicted in Fig. 2. Note that ROIs in medial prefrontal cortex comprised voxels in both hemispheres.

The two-way full factorial ANOVA model implemented in SPM5 was imported into Marsbar, and run using the six ROIs. For each ROI and subject mean cluster beta scores were extracted for each condition. These mean values were used as estimates for the effects of the informative value of the feedback and for feedback valence, using F-contrasts. Effects of the informative value of the feedback were tested by calculating the main effect of feedback group, determining regions more activated by informative compared to non-informative feedback. Effects of feedback valence were calculated within the informative feedback group, by contrasting activation to negative and positive feedback, respectively, which is the contrast that was most often used in fMRI studies focussing on feedback processing. Follow-up tests were then performed on simple effects comparing incorrect performance followed by negative feedback in the informative feedback group with incorrect performance followed by non-informative feedback in the non-informative feedback group and correct performance followed by positive feedback (informative feedback group) vs. correct performance followed by non-informative feedback (non-informative feedback group). A p-value of p<.008 was regarded as significant (Bonferroni corrections for multiple comparisons in the 6 ROIs). For completeness, results of a whole brain analysis are reported in the supplementary material.

Additionally, analyses of brain-behaviour correlations were performed within the informative feedback group, to test the effect of relative frequency of positive and negative feedback on activation in prefrontal ROIs. We correlated subjects’ failure rates, which indicate the amount of negative feedback received, with neural activity related to negative feedback (i.e. the differences scores for trials with errors followed by negative feedback minus trials with correct responses followed by positive feedback). For all analyses, coordinates reported correspond to the standard Montreal Neurological Institute (MNI) brain. Activations are displayed on the single subject template included in SPM5.

To test for possible between-group differences in BOLD-activation in posterior brain regions as an indicator for differences in attention deployment, we used the F-contrast ‘main effect of feedback group’ with a height threshold of p<.001and a cluster threshold of 10 voxels.

### Behavioral Data Analysis

Statistical analysis of behavioral data was performed with SPSS 18 for Windows. A repeated measures analysis of variance (ANOVA) with performance (correct/error) as within-subject factor and feedback group (informative/non-informative feedback group) as between-subject factor was used for the behavioral data obtained in the fMRI session. Reaction times and error rates were used as dependent variables.

To assess if the participants relied on external feedback, we performed a repeated measures ANOVA with error rates (actual/estimated) as within-subject factor and feedback group (informative/non-informative feedback group) as between-subject factor.

## Results

### Behavioural Data

The reaction time data yielded a significant main effect of performance (F_(1,32)_ = 38.88, p<.001), with post-hoc tests showing that reaction times were significantly shorter for correct trials than for error trials in both feedback groups (informative feedback group: 1511±59 msec (mean ± S.E.M), and 1669±66 msec; non-informative feedback group: 1595±61 msec and 1863±83 msec). Importantly, the main effect of feedback group was not significant (F_(1,32)_ = 2.40, p = .131) and no significant differences were found in error rates between the two feedback groups (informative feedback group: 28.9%±2.9%, non-informative feedback group 26.6%±2.8%), ensuring comparableness of brain activation between the two groups. Only.01% of all trials were classified as “too late” (response time >3.5 sec). These trials were excluded from further analyses.

The post scanning questionnaire on estimated error rates indicated that both groups significantly overestimated their actual error rates (mean estimates in the informative feedback group: 42.1%±4.9%, and in the non-informative feedback group 57.7%±3.8%). The discrepancy between actual and estimated errors was significantly higher in the non-informative compared to the informative feedback group (F_(1,32)_ = 12.44, p = .001), indicating that participants were largely dependent on external feedback to assess performance outcomes.

### fMRI Data

#### Informative value of the feedback

At first, the main effect of feedback group (informative/non-informative) was calculated to test for effects of the informative value of the feedback in pre-specified prefrontal ROIs. Subsequently, we performed follow-up tests for the correct and incorrect performance conditions. A significant main effect of feedback group, indicating sensitivity to the informative value, was found in all prefrontal areas tested (p<.005), except for the pre-SMA which only revealed a tendency for significance (F_(1,32)_ = 7.31; p = .009). Descriptively, correct and incorrect responses followed by informative feedback (i.e. positive or negative) yielded higher signal increases as compared to responses followed by non-informative feedback.

#### Incorrect performance

Focussing on trials with incorrect performance, we found significantly increased neural activity in medial and lateral prefrontal brain regions in the informative compared to the non-informative feedback group (see Fig. 2, white bars). In the RCZ: (F_(1,32)_ = 8.55; p<.005), the pre-SMA: (F_(1,32)_ = 9.79; p<.005), bilateral insulae (left: F_(1,32)_ = 36.37; p<.001, right: F_(1,32)_ = 23.64; p<.001) and right IFC (F_(1,32)_ = 13.93; p<.001). In other words, these brain regions exhibited higher neural activity when errors are followed by negative feedback. A similar pattern was seen in the left IFC (F_(1,32)_ = 7.38; p<.01) which did not survive multiple comparisons.

#### Correct performance

Significant between group differences were found bilaterally in the insulae for correct responses followed by positive feedback as compared to correct responses followed by non-informative feedback (see Fig. 2, black bars) (left: F_(1,32)_ = 18.93; p<.001, right: (F_1,32)_ = 15.91; p<.001). A similar pattern was seen in other medial and lateral prefrontal regions such as the RCZ (F_(1,32)_ = 4.71; p<.05) and the IFC (left: F_(1,32)_ = 4.94; p<.05; right: F_(1,32)_ = 6.77; p<.05). These however did not survive correction for multiple comparisons. Note that in the pre-SMA, no significant between group differences (even uncorrected) could be obtained for the correct performance in the between-group comparison (F_(1,32)_ = 1.98; p = .16).

Thus, follow-up testing revealed a considerable signal increase to both negative and positive feedback in bilateral inferior IFC and adjacent anterior insulae, when compared to the respective non-informative feedback condition. Albeit, with respect to the positive feedback condition, only the anterior insulae revealed significant signal increases. Despite some discrepancies across studies, these prefrontal areas have been more often associated with the processing of unfavourable outcome information, which was also evident in our within group comparison (whole brain analysis), showing higher neural activity to negative as compared to positive feedback in the pre-SMA and in bilateral inferior frontal cortex and adjacent insulae (see supplementary Material, Table S1, online).

#### Feedback valence

Effects of feedback valence were tested within the informative feedback group, comparing correct and incorrect responses followed by positive and negative feedback respectively (Fig.2). Significant valence effects, with more activation for negative feedback were obtained in the pre-SMA (F_(1,16)_ = 13.76; p<.001), the ventral anterior insulae (left: F_(1,16)_ = 18.80; p<.001, right: F_(1,16)_ = 14.53; p<.001) and the right IFC (F_(1,16)_ = 10.04; p<.005). A similar pattern was seen for the left IFC (F_(1,16)_ = 6.56; p<.05), but this effect did not survive corrections for multiple comparisons. No valence effect was found in the RCZ (F_(1,16)_ = 0.59; p = .45). The RCZ ROI used here was located in the anterior-most part of the rostral cingulate zone, [24] and is part of the putative homologue of the rostral motor cingulate area (CMAr) in monkeys [25]. As can be seen in Fig. 2, the lack of significant differences in the RCZ is due to increased neural activity for both, correct responses followed by positive feedback and incorrect responses followed by negative feedback. Note that there was no single ROI more activated by positive compared to negative feedback.

#### Brain-behavior correlations

**Figure 3:**
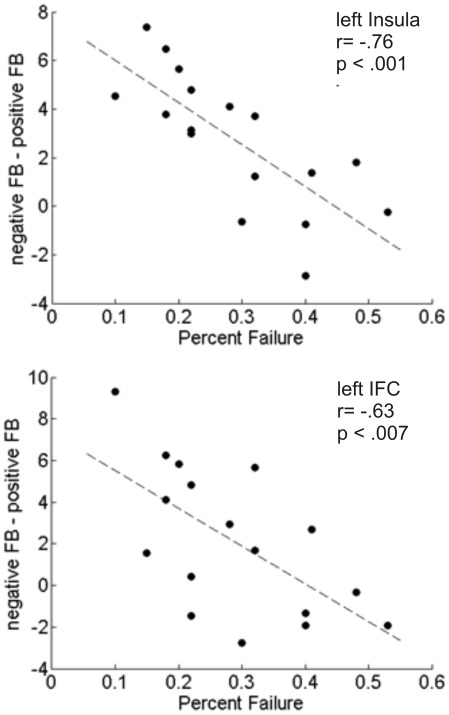
Brain-behaviour correlations in the insula and inferior frontal cortex. Correlation of subjects’ failure rates with the difference scores obtained from activation to both feedback values (ROI value negative feedback – ROI value positive feedback). Results of the correlation analyses are depicted for the left insula and inferior frontal cortex IFC. FB = feedback.

It has been hypothesized that neural activity in medial prefrontal cortex may depend on the frequency of positive and negative feedback (Nieuwenhuis et al., 2005). To investigate the influence of feedback frequency on neural activity in prefrontal cortex, we correlated subjects’ failure rates, which indicate the amount of negative feedback received, with neural activity related to negative feedback (i.e. difference scores for negative vs. positive feedback). Note that this analysis can only be performed in the group receiving informative, i.e. positive and negative feedback. The analysis revealed that failure rates were negatively correlated with difference scores in left lateral prefrontal regions (Fig. 3), including the left IFC (r = .63, p = .007) and the left anterior insula (r = .76, p<.001). In the case of the left insula, this was due to the expected pattern that less failures were associated with higher activation to negative feedback (r = .636, p = .006). No significant correlations were obtained for the RCZ (r = .098, p = .709) and pre-SMA (r = .10, p = .703).

## Discussion

The present study aimed at investigating the contribution of prefrontal brain regions to the processing of informative feedback (informative vs. non-informative feedback) and feedback valence (positive vs. negative feedback), when feedback does not contain task-specific information to improve performance in the following trial. We show that even under these conditions the RCZ, in contrast to other prefrontal regions, is not sensitive to feedback valence, with similar signal increases to both negative and positive informative feedback. In contrast, the pre-SMA, IFC and insula were sensitive to both the informative value and the valence aspects of the feedback. All valence-sensitive prefrontal areas revealed increased activation to negative compared to positive feedback.

### ACC/RCZ Involvement in Positive and Negative Feedback

Despite an overwhelming evidence for the more posterior ACC region as the generator of the error and feedback related negativity (ERN, FRN), heightened neural activation related to negative compared to positive feedback has been observed only in some fMRI studies investigating feedback processing [3], [6], [9], [26]–[27]. In others, as in the present one, no significant differences were apparent between the two conditions in the dACC/RCZ [1]–[2], [4], [28]. For between-group comparisons (informative vs. non-informative feedback), we observed a considerable signal increase in the RCZ to both positive and negative feedback. This finding is also supported by the whole brain analyses depicted in Tables S1 and S2 of the supplementary material. Here, significant activation for the RCZ was obtained for both correct and incorrect performance followed by informative feedback when compared to the respective non-informative feedback condition. No significant activation in the RCZ was obtained when comparing activation to negative and positive feedback in the informative feedback group. Lack of a simple valence-specifity of the ACC/RCZ has been supported from many monkey electrophysiology studies and there is plenty of evidence for the homologue of the ACC/RCZ in particular to use both favourable and unfavourable value information, e.g. to integrate reward history, evaluate outcomes and select appropriate actions with regard to relevant contextual information [29]–[31].

In humans, subsequent to the extremely fruitful and stimulating research on ERP components elicited by unfavourable outcomes, earlier EEG and fMRI studies on ACC functions have largely emphasized a role of this brain region in error and conflict detection (for a review see [32]). In more recent studies with humans, the involvement of the dACC/RCZ and other medial and lateral prefrontal areas in positive besides negative feedback evaluation has received increased attention and evidence is now accumulating for a strong impact of behavioral relevance on feedback-induced neural activity in the RCZ [15], [18]. In our task, positive and negative feedback were of similar relevance in eliminating uncertainty related to performance outcomes and similar activation increases were obtained for both positive and negative feedback when compared to the respective non-informative condition. As five novel picture-pseudoword associations, presented in rapid succession, had to be maintained for retrieval within each trial, errors were hard to detect and participants heavily relied on external feedback. This was underscored by the marked overestimation of error rates, shown to be significantly higher in the non-informative compared to the informative feedback group. The relatively higher BOLD signal increase to the negative-feedback condition is possibly due to the fact that this outcome, in a proportion of trials, may have additionally signaled demands for more attention resources and thus bore an additional informational value.

An impact of the behavioral relevance of feedback on dACC/RCZ activation was already observed in early fMRI studies with humans [17], [20]. Based on these studies and on findings from monkey electrophysiology, it has been claimed that evidence pointing to a dominant role of the ACC for negative outcomes may partly derive from experimental biases, i.e. from a non-equivalence of the behavioural relevance [13]. Two recent fMRI studies, using an adaptive time estimation task, added further relevance to this assumption. In a study by van der Veen, et al. [11], where positive feedback was assumed to give a more informative clue on how to perform the task, more activation to positive as compared to negative feedback was obtained in a ROI analysis for the RCZ. Interestingly, in a second study by Mies et al. [18], a minor change in the feedback presentation, most likely leading to a comparable relevance of both positive and negative feedback, resulted in a similar signal increase to both feedback types. Importantly, results of these studies, together with our findings – in a completely different paradigm -, challenge the assumptions of the reinforcement theory, which predicts more RCZ activation when the outcome of behaviour is worse than expected. Hence, it has been suggested that the RCZ evaluates whether feedback is relevant or not, providing the opportunity to fine tune upcoming behaviour [18].

Overall, in previous studies showing comparable signal increases to both positive and negative feedback in the RCZ of humans, both feedback types were equally relevant and feedback could be used for behavioural adjustments in subsequent trials [17]–[18], [20]. This is well in accordance with a role of the RCZ in forming and continuously updating action-outcome associations to optimally adapt behaviour [33]. However, it cannot be excluded that a similar activation to positive and negative feedback in the RCZ is substantially linked to the potential usefulness for upcoming performance. We here show that positive and negative feedback both activate the RCZ even though the feedback could not be used to adapt performance in the next trial (apart from a general increase in attention to task requirements in the case of negative feedback). Unlike the recently published study by Mies et al. [18], who were the first to explicitly focus on neural effects of valence and validity of feedback by using feedback of equal relevance, our task that did not allow for task-specific performance adjustments. Further studies should be conducted to directly compare feedback with and without information relevant for task-specific adjustments in the following trials or feedback that only gradually differs in the amount of adjustments that it allows for.

### The Pre-SMA is Primarily Associated with Negative Feedback

Increased activation of the pre-SMA for negative compared to positive feedback has been reported in some studies, albeit mostly ascribed to other processes like to events preceding the feedback [2], response-related processes [26] or uncertainty going along with errors in general [3]. Note that the behavioural data in our study also provides evidence for higher uncertainty and pre-response conflict in case of an incorrect response, with significantly slower mean reaction times in both the informative and non-informative feedback groups for incorrect compared to successful performance. However, contrasting error trials with informative vs. error trials with non-informative feedback (between groups) prevented the confounding influence from ongoing processes related to task performance, as the degree of response uncertainty and pre-response conflict should not differ among these events. Thus, by subtracting out activation evoked by the incorrect performance condition of the non-informative feedback group, we were able to show that the responsiveness of the pre-SMA to negative feedback is not related to uncertainty or pre-response conflict. Note that pre-SMA was the only prefrontal site in our study where, when compared to the respective non-informative condition, no signal increase to positive feedback was observed (even at the uncorrected level). Therefore we assume that heightened pre-SMA activation found in our study is clearly and specifically associated with negative feedback processing. This finding is in accordance with the proposed role of the pre-SMA in error detection [34], but also with its role in performance monitoring and conflict detection [35]. It is well conceivable that activation of the pre-SMA in our study is related to error detection and/or post-response conflict between actual and expected or hoped-for feedback values.

### Failure Rates are Correlated with Neural Activity in Inferior Frontal Cortex and Insulae

Experiments with non-human primates revealed that midbrain dopamine neurons code the discrepancy between actually obtained and predicted rewards, with increased (decreased) firing rates when the outcome is better (worse) than expected [36]–[37]. These bidirectional phasic responses of dopamine neurons have been proposed to convey a teaching signal to regions implicated in reward-related learning, such as the ACC and the basal ganglia [38] and thus to serve flexible behavioural adjustments and guidance of future learning. If failures are less frequent, negative feedback is less expected, particularly in tasks where errors are hard to detect. It has been shown that less frequent negative feedback was associated with a larger feedback ERN [39] and in imaging studies which reported increased ACC activation for negative vs. positive feedback, errors were less frequent than successes [3], [6], but see Cools [28]. In contrast, in studies showing a lack of differential ACC activation for negative as compared to positive feedback, both feedback types occurred with equal frequency [2], [4], but see results of the current study and Holroyd [9]. Based on these results, it has been suggested that part of the variability of ACC activity can be explained by the relative frequency of positive and negative outcomes [2], [40]. To assess the effect of outcome frequency on recruitment of prefrontal ROIs, we correlated individual subjects’ failure rates (i.e. the individual amount of negative feedback) with their respective activation differences between positive and negative feedback. Based on previous data we expected that higher failure rates (up to approximately 50%) are associated with smaller activation differences. While we found no significant relationship between failure rates and neural activity in the medial prefrontal cortex (RCZ and pre-SMA), the left ventrolateral PFC (insula and IFC) revealed a significant negative correlation between failure rates and activation differences between negative and positive feedback. In other words, the less the errors a subject made, the higher the difference in activation between both outcomes in these areas (Fig.3).

Apart from several target areas of the mesocortical and mesolimbic dopaminergic systems the insula has also been shown to be responsive to prediction errors [41]–[43], corroborating its assumed relevance for loss related learning. However a recent study suggests that insula activity may be better explained with its role in saliency detection rather than detection of prediction errors [44]. If failures are less frequent they are also less expected and at the same time more salient, bearing an aspect of novelty. Hence, it seems possible that less frequent negative outcomes in our study led to a relatively strong signal increase compared to positive outcomes. These findings are well in line with the notion that the insula and frontal operculum are part of a larger network that serves to identify salient features in the environment [45]. Downar and colleagues could show significant activation increases in the insula to task-relevant compared to task irrelevant changes [46] and in the right insula and IFC for novel compared to familiar stimuli [47]. Though the anterior insula has been linked to emotionally salient stimuli, its functional significance has mostly been associated with the processing of aversive emotions [48]. Several fMRI studies have demonstrated that the anterior insula and IFC are more activated in incorrect as compared to correct trials [49] and by negative as compared to positive feedback [3], [5], [10]. Our finding of significantly increased activation to both negative and positive feedback (compared to the respective non-informative condition) corroborate findings from previous studies indicating that the anterior insula is responsive to both negative and positive emotional stimuli [15]–[16]. The correlation analysis further suggests that differential neural activity to negative and positive feedback may be due to the relative frequency of those events.

### Limitations of the Study

A notable limitation of the study included the use of two different groups, one for the informative and another for the non-informative feedback condition. The use of a non-informative feedback condition in studies with immediate feedback is clearly beneficial to avoid confounds from on-going processes related to task performance. On the other hand, using a separate group performing with non-informative feedback as a control bears the risk of confounds originating from uncontrolled between-group differences. To minimize the effects of possible group differences we ensured comparability in key variables like age and intelligence. It should be mentioned that mixing trials with informative and non-informative feedback may also have disadvantages. In runs with mixed trials, there is a certain expectation to receive outcome-related information compared to runs with non-informative feedback only. Thus, receipt of non-informative feedback can be felt as an omission of outcome information, possibly resulting in heightened uncertainty.

### Conclusion

This study is the first to explicitly focus on effects of informative feedback and feedback valence when feedback bears no task-specific information for subsequent performance. As with previous studies that used equally relevant positive and negative feedback, involving very different paradigms, and a type of feedback that was relevant for task-specific behavioural adjustments, the RCZ was not sensitive to the valence of the feedback. This suggests that independent of the paradigm and type of feedback (enabling vs. not enabling task specific adjustments), the RCZ exhibits valence-insensitivity, whereas other prefrontal areas do not show this pattern. We were further able to show, that in our task, some prefrontal regions, which have often been associated with the processing of negative feedback, were also involved in processing positive feedback. The only exception was the pre-SMA, which was shown to be specifically associated with negative feedback processing. Thus, when feedback lacks information needed for performance adjustments, the pre-SMA may be primarily involved in processing unfavourable outcome information necessitating an increase in cognitive control and heightened attention, whereas the RCZ, as the more cognitive part of the ACC may be more involved in evaluation of both positive and negative outcomes.

## Supporting Information

Table S1 Whole brain analyses: Within-subject contrasts comparing activation to positive and negative feedback in the informative feedback group.
(DOCX)

Table S2 Whole brain analyses: Between-subject contrasts for correct and incorrect performance with informative and non-informative feedback, respectively.
(DOCX)
