# Supplementary material for: Prefrontal Neural Activity When Feedback Is Not Relevant to Adjust Performance
Source: PLoS One. 2012 May 16;7(5):e36509. doi: 10.1371/journal.pone.0036509 (PMC3353938; doi:10.1371/journal.pone.0036509)
Supplement: Table S1 — Whole brain analyses: Within-subject contrasts comparing activation to positive and negative feedback in the informative feedback group. (DOCX) [file pone.0036509.s001.docx]

**Prefrontal neural activity when feedback is not relevant to adjust performance**

**Online Supplement 1**

**Table S1:** Whole brain analyses: Within-subject contrasts comparing activation to positive and negative feedback in the informative feedback group.

| **Region** | **Brodmann**  **Area** | | **Cluster size** | **MNI coordinates** | | | **z-value** |
| --- | --- | --- | --- | --- | --- | --- | --- |
|  |  |  |  | **x** | **y** | **z** |  |
| **Informative Feedback: Positive > Negative** | | | | | | | |
|  | | | | | | | |
| **Middle Occipital** | 18 | R | 36 | **30** | **-68** | **34** | 3.97 |
| **Putamen** |  | L | 21 | **-30** | **-6** | **2** | 3.95 |
| **Cerebellum** |  | R | 94 | **14** | **-72** | **-14** | 4.10 |
| **Cerebellum** |  | L | 29 | **-16** | **-64** | **-20** | 4.15 |
| **Informative Feedback: Negative > Positive** | | | | | | | |
| **Pre-SMA** | 6 | R | 322 | **6** | **18** | **48** | 4.99 |
| **Inferior frontal/Insula** | 47 | R | 302 | **44** | **28** | **-8** | 4.37 |
| **Inferior frontal/Insula** | 47 | L | 329 | **-34** | **26** | **0** | 5.27 |
| **Middle temporal** | 21 | R | 25 | **50** | **-32** | **-2** | 3.59 |

SMA=supplementary motor area
