# Supplementary material for: Prefrontal Neural Activity When Feedback Is Not Relevant to Adjust Performance
Source: PLoS One. 2012 May 16;7(5):e36509. doi: 10.1371/journal.pone.0036509 (PMC3353938; doi:10.1371/journal.pone.0036509)
Supplement: Table S2 — Whole brain analyses: Between-subject contrasts for correct and incorrect performance with informative and non-informative feedback, respectively. (DOCX) [file pone.0036509.s002.docx]

**Prefrontal neural activity when feedback is not relevant to adjust performance**

**Online Supplement 2**

**Table S2:** Whole brain analyses: Between-subject contrasts for correct and incorrect performance with informative and non-informative feedback, respectively.

| **Region** | **Brodmann**  **Area** | | **Cluster size** | **Talairach Coordinates** | | | **z-value** |
| --- | --- | --- | --- | --- | --- | --- | --- |
|  |  |  |  | **x** | **y** | **z** |  |
| **Correct Performance: Informative vs. Non-informative** | | | | | | | |
|  | | | | | | | |
| **Rostral cingulate zone** | 24 | R/L | 41 | **0** | **32** | **28** | 3.63 |
| **Inferior frontal/Insula** | 47 | R | 107 | **36** | **24** | **-10** | 3.73 |
| **Inferior frontal/Insula** | 47 | L | 80 | **-34** | **20** | **-12** | 4.61 |
|  |  |  |  |  |  |  |  |
| **Incorrect Performance: Informative vs. Non-informative** | | | | | | | |
| **Rostral cingulate zone** | 24 | R/L | 133 | **0** | **30** | **26** | 3.77 |
| **Pre-SMA** | 6 | R | 44 | **10** | **6** | **66** | 3.93 |
| **Pre-SMA** | 8 | R | 75 | **6** | **16** | **52** | 4.06 |
| **Inferior frontal/Insula** | 47 | R | 407 | **36** | **16** | **-10** | 5.19 |
| **Inferior frontal/Insula** | 47 | L | 287 | **-34** | **18** | **-10** | 5.32 |
| **Middle frontal** | 10 | R | 50 | **30** | **56** | **26** | 3.74 |
| **Superior frontal** | 8 | R | 95 | **16** | **44** | **42** | 4.48 |
| **Inferior temporal** | 21 | R | 184 | **58** | **-20** | **-16** | 4.77 |
| **Middle occipital** | 19 | L | 39 | **-32** | **-94** | **2** | 3.73 |
| **Caudate Nucleus** |  | R | 44 | **10** | **6** | **2** | 3.77 |
| **Ventral tegmental** |  | R | 44 | **6** | **-12** | **-10** | 4.58 |
| **Midbrain** |  | R | 74 | **8** | **-28** | **-12** | 4.71 |

SMA=supplementary motor area
